# Early reduction in gut microbiota diversity in critically ill patients is associated with mortality

**DOI:** 10.1186/s13613-024-01407-x

**Published:** 2024-11-26

**Authors:** Hannah Wozniak, Nadia Gaïa, Vladimir Lazarevic, Christophe Le Terrier, Tal Sarah Beckmann, Eleonora Balzani, Martin Urner, Jérôme Pugin, Jacques Schrenzel, Claudia-Paula Heidegger, Gut Microbiota working group, Lorin Fröhlich, Tommaso Rochat Negro, Tania Soccorsi, Noémie Suh, Aurélie Perret, Chiraz Chaabane

**Affiliations:** 1 Intensive Care Unit, Department of Acute Medicine, Geneva University Hospitals, Geneva, Switzerland; 2 Interdepartmental Division of Critical Care Medicine, University of Toronto, Toronto, Canada; 3 Genomic Research Laboratory, Department of Medicine, Geneva University, Geneva, Switzerland; 4 Department of Medicine, Emerging Antibiotic Resistance Unit, Medical and Molecular Microbiology, University of Fribourg, Fribourg, Switzerland; 5 Department of Anesthesiology & Pain Medicine, University of Toronto, Toronto, Canada

**Keywords:** Critical care, Gut microbiota, Patients’ outcome

## Abstract

**Background:**

Critical illness is associated with an altered gut microbiota, yet its association with poor outcomes remains unclear. This study evaluates the early gut microbiota diversity changes in intensive care unit patients and its association with mortality. Additionally, it explores fecal pH as a potential biomarker for these changes.

**Methods:**

In this prospective observational cohort study, fecal samples were collected at two time points: S1, the first stool passed upon intensive care unit admission, and S2, the first stool passed at least 24 h after S1. Full-length 16S rRNA gene sequencing was performed for gut microbiota analysis, with α-diversity measured using the Shannon index. Bayesian joint models were used to estimate the association between time-varying changes in gut microbiota diversity and 60-day mortality, as well as the association between daily changes in stool pH and in diversity.

**Results:**

Twenty-four of 96 patients overall died during follow-up. Daily Shannon index decreased on average by −0.1 points [95% Credible Intervals (CrI) −0.20 to −0.10]. Every point decrease in Shannon index was associated with a 1.99-fold increase in the hazard of death (95% CrI, 1.04 to 4.51). Time-varying fecal pH levels were not associated with changes in Shannon index.

**Conclusions:**

Gut microbiota diversity decreased over time, associated with increased mortality. Fecal pH is an unreliable marker of gut microbiota change. Future studies on gut microbiota and related biomarkers should focus on the initial days in the intensive care unit to detect and mitigate a decline in gut microbiota diversity.

**Supplementary Information:**

The online version contains supplementary material available at 10.1186/s13613-024-01407-x.

## Introduction

Gut microbiota (GM) has a symbiotic relationship with its human host, actively contributing to proper organ function and enhancing overall health [1, 2].

Critical illnesses have been associated with significant disruptions in commensal GM, allowing the proliferation of potentially pathogenic bacteria, a phenomenon referred to as dysbiosis [3]. Although previous research has provided limited insights into the dynamic changes of GM during intensive care unit (ICU) stays, it is now clear that GM experiences considerable fluctuations in ICU patients [3–7].

The association between GM and critically ill patients’ outcomes remains unclear. Several studies have reported a potential association between specific GM taxa or reduced α-diversity and an increased risk of death in all critically ill patients as well as in specific patients groups such as neurocritical care patients [5, 8–11]. However, conflicting results exist, with a recent systematic review failing to establish such association [12–14]. The disparities in previous studies results can, in part, be attributed to the fact that many studies focused on a single time point and did not capture the dynamic changes in GM. It is possible that changes, more than absolute values, matter to assess GM dysfunction. Moreover, the diversity of methods used to assess GM, which spans from examining Bacteroidetes/Firmicutes (B/F) ratios to α-diversity indices, can contribute to the overall inconsistency in the findings and the challenge for establishing definitive evidence. It is currently unclear whether dysbiosis can be considered a form of organ dysfunction associated with mortality. Gaining an understanding of the dynamics of GM changes and its possible consequences is imperative.

Furthermore, because of its cost and complexity to study, the use of GM in clinical practice is challenging. The absence of a readily discernible biomarker to monitor GM changes has hindered previous research efforts. Some studies have suggested that the presence of extreme stool pH values may serve as a surrogate marker associated with particular GM profiles in septic patients [4, 15]. Certain bacterial taxa are influenced by pH levels, with extreme pH conditions leading to a reduction in commensal flora. This reduction, in turn, results in a shift towards a more alkalotic environment due to the decreased production of short-chain fatty acids [15]. However, these findings were based on sporadic measurements and did not provide conclusive insights into the dynamic changes of stool pH and GM. It remains unclear if dynamic changes of stool pH represent a cost-effective and readily available surrogate marker of GM change.

In the present work, we recorded diversity differences within the ICU population, modeled diversity changes over time through the Shannon index—a simple α-diversity index, measured the association between a daily change in Shannon index and mortality up to 60 days, and investigated if fecal pH changes may serve as a practical surrogate marker for GM alterations.

## Methods

### Study design and patients

This study was a prospective observational cohort study conducted in the medico-surgical ICU at the Geneva University Hospital between July 2021 and May 2022. Patients were screened daily and were eligible if they were admitted to the ICU with the following inclusion criteria: age ≥ 18 years, admitted to the ICU in the last 24 h, Acute Physiology and Chronic Health Evaluation (APACHE) II score ≥ 20 points or Simplified Acute Physiology Score (SAPS) II score ≥ 38 points, estimated ICU length of stay (LOS) of ≥5 days (with the 5-day estimation relying on the clinical judgment of the screening clinician). Exclusion criteria included any antibiotic received in the month preceding ICU admission, a history of chronic intestinal inflammatory disease, or gut surgery within the last 3 months. These last exclusion criteria assumed that these factors could greatly affect the initial GM composition, and subsequent modifications might be too subtle to be reliably assessed. Consent to participate in the study was obtained from the patient or from their surrogate decision maker when the patient lacked the capacity to make decisions. For patients who survived and regained decision-making capacity during the follow-up period, retrospective consent was sought. Patient data were prospectively collected in the medical chart and patients were followed until death or reaching 60 days (D-60) after ICU admission. Patients, who were discharged from the Geneva University Hospitals before collecting any stool sample, were excluded of the analysis. The primary outcome was all-cause mortality up to 60 days. We examined the exposure to two specific variables: the Shannon index (α-diversity) and stool pH.

The study was conducted in accordance with the Declaration of Helsinki and received approval on May 2021 from the Geneva Ethical and Research Committee (BASEC-2021-00315). This article followed the STROBE guidelines for reporting results based on observational studies [16].

### Gut microbiota analysis, 16S rRNA sequencing and pH analysis

#### Collection of fecal samples and pH analysis

Samples were obtained at the following two time points: the first stool passed by the patient after ICU admission was collected and labeled as S1 (Stool 1), while the second stool sample was obtained when the patient had passed at least 24 h after S1, and labeled as S2 (Stool 2). Given the unpredictable timing of patients’ bowel movements, specific dates of stool collection were recorded. The initial stool sample (S1) after ICU admission reflects the patient’s GM prior to any ICU-related changes, regardless of the timing, as no stool has been passed in the ICU before S1. Fecal pH in each stool sample were determined in the ICU by one of the investigators, using the same pH meter (HI99161, Hanna Instruments, Langnau, Switzerland) by immersing it into the stool sample for 20 s. Fecal samples S1 and S2 were then collected using DNA/RNA Shield Fecal Collection Tubes (Zymo Research, Irvine, USA) following the manufacturer’s instructions. Obtained suspensions were stored to the research laboratory at −80 °C until DNA extraction.

#### Gut microbiota analysis

DNA extractions were prepared from 250 µL of each stool suspension in DNA/RNA Shield, using the ZymoBIOMICS DNA Miniprep kit (Zymo Research) which included a 15-min bead-beating step. Water (50 µL) was used for DNA elution. In the two negative controls for DNA extraction, 250 µL of DNA/RNA Shield were used instead of a stool suspension. Purified DNA was quantified using the Qubit fluorometer 2.0 with Qubit dsDNA BR and HS Assay Kits (Thermo Fisher Scientific, Waltham, USA) and stored at −20 °C.

One hundred nanograms (ng) of DNA in a 15 µL volume were sent to Genesupport SA Fasteris NGS services, located in Plan-les-Ouates, Switzerland, for full-length 16S rRNA gene (V1–V9) amplification via PCR (with 27F 5′-AGRGTTYGATYMTGGCTCAG-3′/1492R 5′-RGYTACCTTGTTACGACTT-3′ primer pair[17]), barcoding, and subsequent amplicon sequencing. The HiFi sequencing was performed on a PacBio Sequel IIe instrument using a SMRTbell prep kit 3.0 for library preparation, a Sequel II Binding Kit 3.1 for sequencing preparation, and a Sequel II sequencing kit 2.0 along with a SMRT Cell 8 M for sequencing. PacBio sequencing generated 4384 to 47,438 reads per sample (median 17,143) with a mean length of 1464–1522 bp (median 1493) and a mean quality score of Q29–Q40 (median Q32). Sequencing data are available on the European Nucleotide Archive Database under the study accession number PRJEB68229.

Bedtools v.2.27.1 bamtofastq conversion utility was used [18] to extract FASTQ records from sequence alignments in BAM format. A homemade script was used to remove 16S rRNA primer sequences from reads and to distinguish between reads in the forward orientation and those in the reverse orientation with respect to the 16S rRNA gene sequence. ‘Reverse reads’ were reverse-complemented using seqtk v.1.2-r102-dirty (https://github.com/lh3/seqtk).

The reads were clustered into zero-radius operational taxonomic units (zOTUs) using UNOISE3 from USEARCH v.11.0.667 pipeline [19, 20]. We removed zOTUs that exhibited less than 90% identity or matched less than 99% of the length of reference 16S rRNA sequences from the EzBioCloud 16S database [21] using USEARCH. zOTUs were classified using the EzBioCloud 16S database via mothur (method = Wang, cutoff = 80) [22].

Ecological indices (richness and Shannon index [23]) were calculated from the relative abundance of zOTUs after rarefaction to the same sequencing depth (4000 reads per sample) using the *rrarefy* function in the vegan v2.6-2 R v4.2.0 package.

### Statistical analysis

Patients’ characteristics at baseline were reported stratified by mortality using proportions for categorical variables and median (interquartile range, IQR) for continuous variables.

To assess the significance of differences in bacterial communities between groups defined by survival status, we performed a PERMANOVA [24] test with 9999 permutations using PRIMER (PRIMER-e, Auckland, New Zealand). This test was based on Bray–Curtis similarity [25] and square-root transformed relative abundances of bacterial taxa (zOTU, species and genera). Hierarchical clustering of samples using the average linking method was conducted on the Bray–Curtis similarity matrix, derived from the untransformed relative abundances of the 25 most abundant genera. We chose the genus level to reduce data complexity and enhance the overall representation of microbial taxa in visualizing bacterial communities. To assess the relative abundance of taxa in relation to D-60 survival status, a MaAsLin2 [26] algorithm was used in LM mode, applying log transformation, a minimum prevalence 25% and a minimum relative abundance threshold of 0.01%. Benjamini–Hochberg corrected *p*-values < 0.05 were considered significant.

We used Bayesian joint models for longitudinal and time-to-event outcomes to measure the association between daily changes in Shannon index and mortality up to 60 days. Bayesian joint models establish conditional independence between longitudinal and time-to-event outcomes using a latent, subject-specific random effects structure, thereby accounting for informative censoring due to death and adjusting for potential confounding from changes in disease severity over time [27, 28]. Antibiotic administration from ICU admission to the second timepoint, as a time-varying covariate, was included in the joint model [12, 29]. First, a parsimonious model was implemented, and subsequently, joint models were also adjusted for important prognostic baseline covariates, including the reason for ICU admission, the SAPS II score, and the Charlson comorbidity index [10, 30]. Results were presented as hazard ratios (HR) with corresponding 95% credible intervals (CrIs). The analysis was conducted using a Bayesian statistical framework. *p*-values, therefore, represent the tail probabilities of containing the no effect value (i.e., hazard ratio of 1). Finally, Bayesian joint models were used to measure the association between daily changes in stool pH and diversity change (as measured by the Shannon index). All statistical analysis were performed with R version 3.5.3.

## Results

### Patients’ characteristics

Of 821 screened patients, 116 patients met the inclusion criteria (Figure E1, Additional file 1). A total of three patients withdrew participation, 12 died or were transferred out of the hospital before any stool sample was collected, and five encountered technical constraints related to next-generation sequencing. Among the 96 patients included in the final analysis, 24/96 (25%) died before D-60, with two of them before having a S2. Patient characteristics stratified by mortality are illustrated in Table 1. The patients had a median age of 60 years (IQR, 49–73) and 37/96 (38.5%) of them were women. Main reasons for ICU admissions were neurological disease (31/96, 32.3%). The median time between ICU admission and S1 was 5 days (IQR, 4–7), and the median time between S1 and S2 was 2 days (IQR, 2–3).

**Fig. 1:**
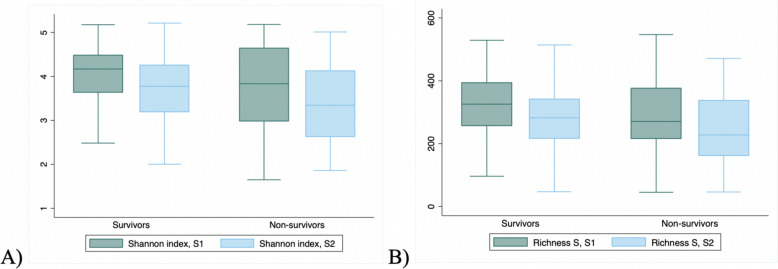
Gut microbiota diversity changes between S1 and S2, according to survivor status. Fig. 1 presents a comparison of gut microbiota Shannon index (**A**) and richness (**B**) between the first (S1) and second (S2) stool sample at the zOTUs level in two distinct groups: survivors and non-survivors. *Boxplots* represent median and interquartile range (IQR) of the Shannon index and of the richness. It indicates a decrease of diversity and richness over time

**Table 1:** Patients’ characteristics according to survival status at D-60

*n* = 96	Survivors *n* = 72	Non-survivors *n* = 24
Sex female, *n* (%)	28 (38.9%)	9 (37.5%)
Age, median (IQR)	58 (45–73)	68 (58–74)
Charlson comorbidity index, median (IQR)	2 (1–5)	5 (3–6)
Reason for ICU admission, *n* (%)		
Septic shock	5 (6.9%)	3 (13.6%)
Cardiovascular disease	13 (18.1%)	8 (33.3%)
Respiratory Insufficiency	13 (18.1%)	5 (22.7%)
Neurological disease	26 (36.1%)	5 (20.8%)
Other^a^	15 (20.8%)	3 (12.5%)
SAPS II on ICU admission, median (IQR)	49 (34–62)	52 (40–73)
APACHE II on ICU admission, median (IQR)	22 (20–26)	26 (20–34)
SOFA on ICU admission, median (IQR)	10 (8–11)	10 (6–12)
Shock on ICU, admission, *n* (%)	31 (43.7%)	15 (62.5%)
CRP (mg/L) on ICU admission, median (IQR)	35.6 (7.4–132.9)	37.6 (4–136.2)
Intubation, n (%)	68 (94.4%)	23 (95.8%)
Time (days) under mechanical ventilation, median (IQR)	11 (6–16)	10 (8–17)
Noradrenaline use between ICU admission and S1, *n* (%)	57 (79.2%)	21 (87.5%)
Noradrenaline use between S1 and S2, *n* (%)	61 (84.7%)	23 (95.8%)
Antibiotics use between ICU admission and S1, *n* (%)	56 (77.8%)	22 (91.7%)
Antibiotics use between S1 and S2, *n* (%)	54 (75%)	22 (91.7%)
Proton pump inhibitors use between ICU admission and S1, *n* (%)	58 (80.6%)	19 (79.2%)
Proton pump inhibitors use between S1 and S2, *n* (%)	61 (84.7%)	21 (87.5%)
Laxatives use between ICU admission and S1, *n* (%)	64 (88.9%)	19 (79.2%)
Laxatives use between S1 and S2, *n* (%)	63 (87.5%)	19 (86.4%)
Enteral nutrition administered between ICU admission and S1, *n* (%)	64 (88.9%)	21 (87.5%)
Enteral nutrition administered between S1 and S2, *n* (%)	66 (91.7%)	20 (83.3%)
ICU length of stay, median (IQR)	16 (9–21)	12 (9–21)
Hospital length of stay, median (IQR)	36 (20–60)	17 (10–37)
Time (days) between ICU admission and S1, median (IQR)	5 (4–7)	5 (4–7)
Time (days) between S1 and S2, median (IQR)	2 (2–3)	2 (2–3)
Shannon index S1 zOTU, median (IQR)	4.2 (3.6–4.5)	3.8 (3–4.7)
Shannon index S2 zOTU, median (IQR)	3.8 (3.2–4.2)	3.4 (2.6–4.1)
Richness S1 zOTU, median (IQR)	325.5 (256–395)	270.5 (215–377.5)
Richness S2 zOTU, median (IQR)	282 (215.5–343)	227.5 (161–339)
Stool pH S1, median (IQR)	6.7 (6.3–7)	6.7 (6.4–6.9)
Stool pH S2, median (IQR)	6.5 (6–7.1)	6.9 (6.4–7.5)

*ICU* intensive care unit, *CRP* C-reactive protein, *S1* first stool sample, *S2* second stool sample

^a^Other: metabolic disorders, kidney failure, abdominal disease, drug abuse, ear-nose-throat surgery

The median SAPS II score was 49 (IQR, 34–62) in survivors and 52 (IQR, 40–73) in non-survivors. Survivors had a median Charlson comorbidity index of 5 (IQR, 3–6) vs. 2 (IQR, 1–5) in non-survivors. When comparing exposures to specific treatments between S1 and S2, antibiotic exposure was noted in 54/72 (75%) of survivors and 22/24 (91.7%) of non-survivors (details on the types of antibiotic used are available in Supplementary Table E1, Additional file 1); laxative treatments were administered to 63/72 (87.5%) of survivors and 19/24 (86.4%) of non-survivors; enteral nutrition was administered to 66/72 (91.7%) of survivors and 20/24 (83.3%) of non-survivors; proton pump inhibitor usage was observed in 61/72 (84.7%) of survivors and 21/24 (87.5%) of non-survivors. The median Shannon-index of the overall population was 4.1 (IQR, 3.6–4.5) at S1 and 3.7 (IQR, 3–4.3) at S2. Survivors presented a median Shannon index of 4.2 (IQR, 3.6–4.5) at S1 and 3.8 (IQR, 3.2–4.2) at S2. Non-survivors had a median Shannon index of 3.8 (IQR, 3–4.7) at S1 and 3.4 (IQR, 2.6–4.1) at S2 (Table 1; Fig. 1).

### Gut microbiota is different among ICU patients

**Fig. 2:**
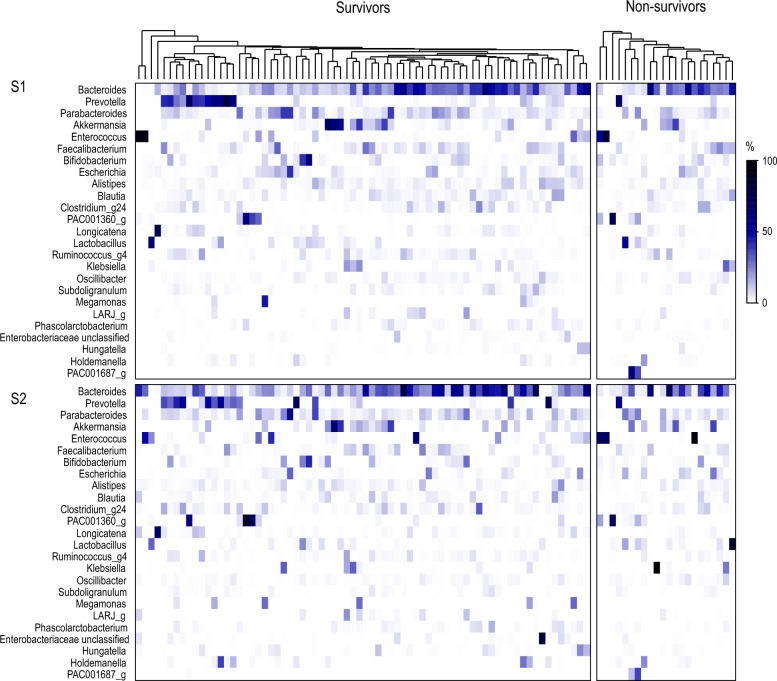
Hierarchical clustering and heat plot of relative abundances of bacterial genera among patients. Fig. 2 illustrates the variation in relative abundances of bacterial genera among patients, with each column representing an individual. The *top panel* displays the hierarchical clustering of S1 samples. The *bottom panel* shows the heat plot for S2 samples, ordered in the same sequence as S1 samples. The figure includes the top 25 genera with the highest mean relative abundance, ranked in descending order. The relative abundance scale is indicated next to the plot. *S1* first stool sample, *S2* second stool sample

The variation in GM composition among patients is depicted in Fig. 2 at the genus level. There was considerable variation in GM composition among patients, even at S1. The results of the PERMANOVA test at the zOTU, species and genus levels indicated no significant overall differences in microbiota composition between survivor and non-survivor patients at either the S1 or S2 sampling points (*p* > 0.05). However, numerous taxa exhibited differential abundance (MaAsLin 2) between survivors and non-survivors at S1 and/or S2 (Supplementary Table E2). After correcting for multiple comparisons, significant differences remained only for genera *Parabacteroides* and *Coprobacter* at S2 (Supplementary Figure E2).

### Change in diversity and its association with mortality

In the Bayesian joint model analysis (Table 2), the Shannon index of patients decreased on average by −0.10 (95% CrI, −0.20 to −0.10; *p* = 0.001) points per day. Every decrease in daily Shannon index was associated with a 1.99-fold increase in the hazard of death (95% CrI, 1.04–4.51; *p* = 0.035), adjusted for baseline SAPS II score, Charlson comorbidity index and reason for ICU admission.

**Table 2:** Bayesian joint model analysis estimating the association between the change in gut microbiota diversity and mortality at day 60

	Estimate (95% credible interval)	*p-*value
Time-to-event		
SAPS II score at ICU admission	1.02 (0.99 to 1.05)	0.26
Charlson comorbidity index	1.14 (0.97 to 1.35)	0.11
Reason for ICU admission		
Sepsis	1.82 (0.22 to 12.25)	0.52
Cardiac failure	2.79 (0.70 to 13.07)	0.15
Respiratory failure	1.95 (0.39 to 9.40)	0.40
Neurological disease	1.65 (0.34 to 9.37)	0.56
Other^a^	Reference	Reference
Decrease in Shannon index (Hazard ratio)	1.99 (1.04 to 4.51)	0.035
Time-varying Shannon index		
(Intercept)	4.30 (4.00 to 4.60)	–
Change per day	−0.10 (−0.20 to −0.10)	<0.01
Change per day * Antibiotics (from admission to S1)	0.00 (0.00 to 0.10)	0.08
Change per day * Antibiotics (from S1 to S2)	0.00 (0.00 to 0.10)	0.16

Sample size = 96; Number of longitudinal observations = 190; Number of events = 24 (25%)

*p*-value represent the tail probabilities of containing the zero-effect value

^1^Other: metabolic disorders, kidney failure, abdominal disease, drug abuse, ear-nose-throat surgery

Exposure to antibiotics did not influence the observed changes in the Shannon index (the posterior probability that the estimate includes the no effect value was 8% from ICU admission to S1 and 16% from S1 to S2, see Table 2). Additional details on the joint model analysis are provided in Supplementary Table E3.

No association between a change in daily fecal pH and mortality was found in the joint model analyses (HR, 1.07; 95% CrI, 0.40–2.26; *p* = 0.796; see Table 3). Also, changes in fecal pH did not reflect changes in the Shannon index (*p* = 0.939).

**Table 3:** Bayesian joint model analysis estimating the association between the change in stool pH, gut microbiota diversity, and mortality at day 60

	Estimate (95% credible interval)	*p*-value
Time-to-event		
pH (Hazard ratio)	1.07 (0.40 to 2.26)	0.796
Time-varying pH		
(Intercept)	6.50 (6.17 to 6.77)	–
Change in pH per day	0.02 (−0.05 to 0.09)	0.554
Change in pH per day * change in Shannon index	0.00 (−0.02 to 0.02)	0.939

Sample size = 96; Number of longitudinal observations = 190; Number of events = 24 (25%)

*p*-value represent the tail probabilities of containing the zero-effect value

## Discussion

A decrease in α-diversity of GM among the first few days of ICU admission, measured by the Shannon index, was associated with an increased risk of death in critically ill patients, adjusted for baseline comorbidities, severity of illness, and the reason for ICU admission. Changes in stool pH, however, did not reflect changes in the Shannon index and were not associated with mortality, indicating that fecal pH is not a reliable surrogate marker of GM diversity changes.

Operating at the zOTU level in our approach offers the advantage of avoiding misclassifications and reliance on taxonomic assignments. Our use of the Shannon index as a continuous variable for α-diversity assessment ensures future replicability. This distinguishes us from studies that relied on specific taxa, on the challenging B/F ratio interpretation, or categorized the Shannon index as low or high, potentially losing valuable information [3–5, 12]. Furthermore, by examining the dynamic changes in GM over time, our study offers comprehensive insights beyond conventional single time point measurements [13]. This sets a new standard for understanding GM dynamics and implications in critical care.

Our study reports a significant decline in GM diversity over time, regardless of the initial GM composition, and its association with mortality at Day 60. Existing literature presents conflicting results on this association. While Lankelma et al.’s pilot study, involving 34 patients, found no association between GM diversity change and in-ICU death [12], other studies have reported such associations [5, 9, 10]. A recent systematic review, encompassing 26 studies, found no clear association between decreasing GM diversity and mortality, and emphasized the shortage of longitudinal studies evaluating GM diversity change and poor outcomes [13]. These studies are limited by small sample sizes, patient population selectivity, limited adjustment for important confounders, and a wide range of GM assessment methods, from changes in relative abundance to the B/F ratio. Our study underscores the importance of going beyond previous research that has primarily focused on single GM measurement at ICU admission and recognizing dynamic alterations in GM during the initial days in the ICU, as a form of organ dysfunction associated with adverse outcomes [8]. Future GM studies should prioritize the early days post-ICU admission, recognizing this period as potentially amenable for interventions that aim to mitigate the decline in GM and might influence patient outcomes.

Interestingly, our study, while including antibiotic-naive patients upon ICU admission, did not observe that antibiotic exposure significantly contributed to the change in the Shannon index during the initial days in the ICU, which contrasts with previous studies [10]. There are several possible explanations for this finding. First, the Shannon index might be mainly influenced by the severity of illness, making the effect of antibiotics in our cohort of critically ill patients relatively minor or negligible. Second, there might be heterogeneity in effects due to different antibiotic classes [31], resulting in an indeterminate total effect of antibiotics on GM changes. Finally, the Shannon index is a diversity index and does not account for the types of bacteria present in the GM composition.

This study does not support the use of stool pH as a substitute indicator for modification of GM diversity. Prior studies in critically ill septic patients found associations between extreme stool pH values and specific GM profiles [4, 15]. However, those research studies collected stool samples at random time points, making it difficult to assess their utility for clinical application. Our research, focusing on the early ICU days, uncovered that changes in α-diversity do not correlate with dynamic pH fluctuations, challenging the use of fecal pH as a reliable GM surrogate. One potential explanation is that pH levels vary considerably throughout the gastrointestinal tract, with a lower pH in the stomach and small intestine and a higher pH in the colon [32]. These shifts can be influenced by intestinal perfusion, nutrition, pharmacological interventions, and disease severity, further complicating the interpretation of stool pH alone. The promising proposal to use stool pH as a direct surrogate marker of GM changes, as suggested by earlier studies, may be compromised by its sensitivity to various confounding variables. Currently, there is a need for a practical, cost-effective surrogate marker for GM, like monitoring urine output for kidney failure or coagulation for liver function. Future studies should prioritize this goal.

Our study has several limitations. First, it has a monocentric design and lacks mycobiota assessment, despite recent research by Prevel et al*.* suggesting an independent association between low mycobiota α-diversity and poor outcomes in critically ill patients [8]. Secondly, the time intervals between ICU admission and the collection of S1, as well as between S1 and S2, varied within the cohort due to individual differences in bowel movements. To address the variability between admission, S1 and S2, we employed a Bayesian joint model, preventing confounding by the timing of stool samples and thereby enhancing the robustness of our results. Additionally, while the use of rectal swabs could have minimized this variation, it would not have allowed us to assess the pH, an important component of our analysis. Thirdly, Bayesian joint models assume that longitudinal and time-to-event outcomes are conditionally independent based on a correctly specified latent, subject-specific random effect structure, thereby accounting for all time-fixed and time-varying confounding. This assumption is strong and implies that all baseline covariates are measured without error, with no residual confounding. However, the absence of residual confounding cannot be tested in observational studies. Lastly, while we adjusted for key baseline prognostic variables and antibiotic exposure, we did not investigate the specific influence of other factors, such as immune function or dietary interventions on GM changes. This could be further investigated in future studies to provide a more comprehensive understanding of their impact on GM dynamics.

## Conclusions

Critically ill patients present significant heterogeneity in their GM upon ICU admission. Regardless of the initial GM composition, reasons for ICU admission, disease severity, comorbidities, and antibiotic administration, a consistent reduction in GM diversity over time was observed, associated with an increased risk of death. Importantly, stool pH did not appear as a reliable surrogate marker for GM diversity changes. Future studies on GM and related biomarkers should prioritize the early days following ICU admission, recognizing this period as potentially amenable for interventions that aim to mitigate the decline in GM and might influence patient outcomes.

## Supplementary Information

Additional file 1.

## Data Availability

The datasets used and/or analysed during the current study are available from the corresponding author on reasonable request.

## Abbreviations

GM: Gut microbiota
ICU: Intensive care unit
