# Supplementary material for: Early reduction in gut microbiota diversity in critically ill patients is associated with mortality
Source: Ann Intensive Care. 2024 Nov 26;14:174. doi: 10.1186/s13613-024-01407-x (PMC11599525; doi:10.1186/s13613-024-01407-x)
Supplement: Supplementary file 1 — Additional file 1. [file 13613_2024_1407_MOESM1_ESM.docx]

**Additional file**

**Early reduction in gut microbiota diversity in critically ill patients is associated with mortality**

Table of contents

Fig. E1.......................................................................................................................2

Supplementary Table E1...........................................................................................3

Supplementary Table E2...........................................................................................3

Fig. E2.......................................................................................................................4

Supplementary Table E3...........................................................................................5

**Figure E1. Study flowchart**

**
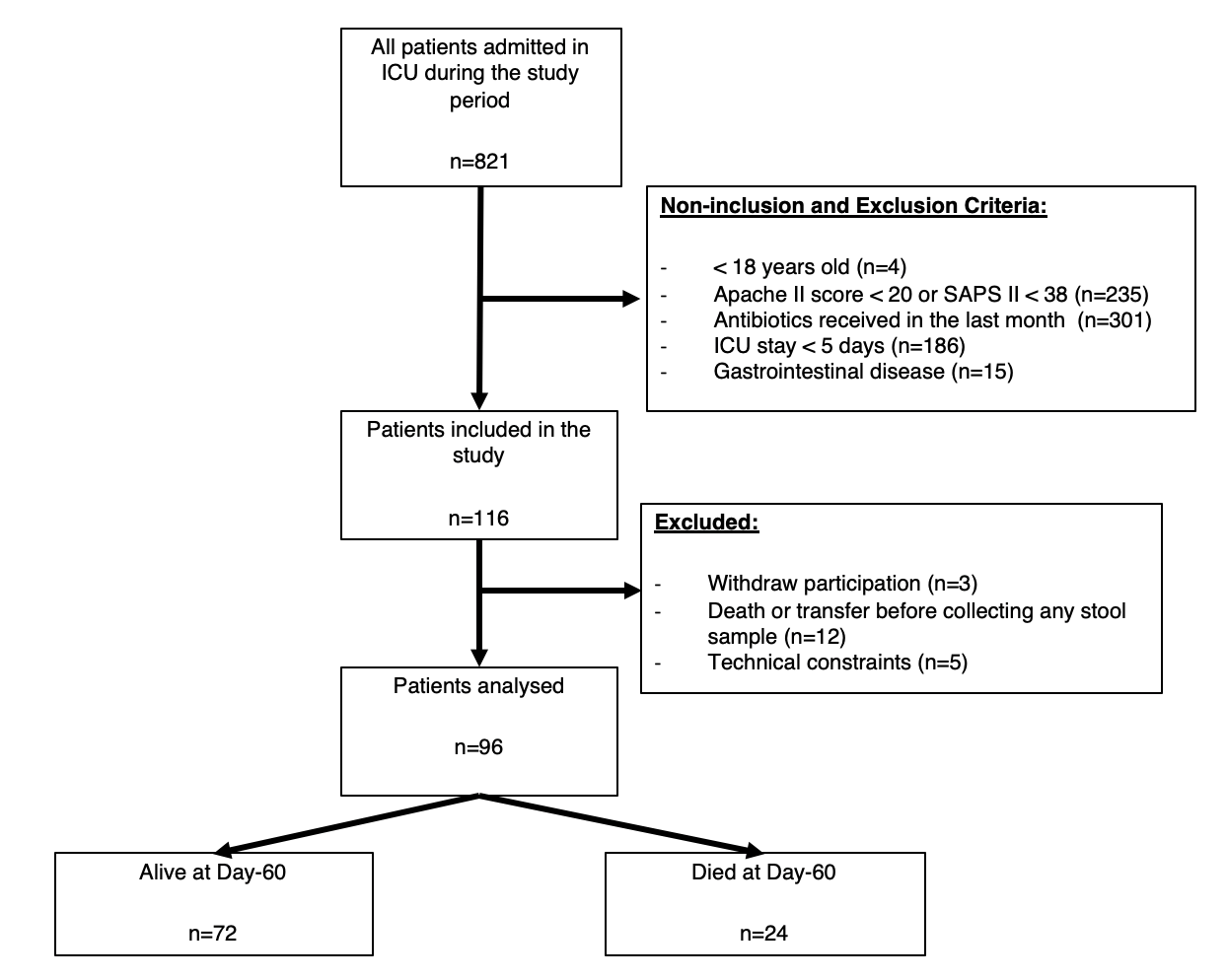
**

**Supplementary Table E1. Antibiotics exposure from ICU admission to S2 in the overall cohort**

| Antibiotic Class | Example of Antibiotics Used | *n*=96 |
| --- | --- | --- |
| Penicillin | Co-amoxicillin, Piperacillin-Tazobactam | 84 (87.5%) |
| Cephalosporins | Cefuroxime, Ceftriaxone, Cefepime | 18 (18.8%) |
| Carbapenems | Meropenem, Imipenem | 11 (11.5%) |
| Fluoroquinolones | Levofloxacin, Ciprofloxacin | 5 (5.2%) |
| Aminoglycosides | Amikacin | 4 (4.2%) |
| Sulfonamides | Trimethoprim-Sulfamethoxazole | 3 (3.1%) |
| Macrolides | Clarithromycin | 9 (9.4%) |
| Lincosamide | Clindamycin | 2 (2.1%) |
| Nitroimidazoles | Metronidazole | 6 (6.3%) |
| Glycopeptides | Vancomycin | 14 (14.6%) |

**Supplementary Table E2. Bacterial taxa showing differential abundance between survivors and non-survivors**


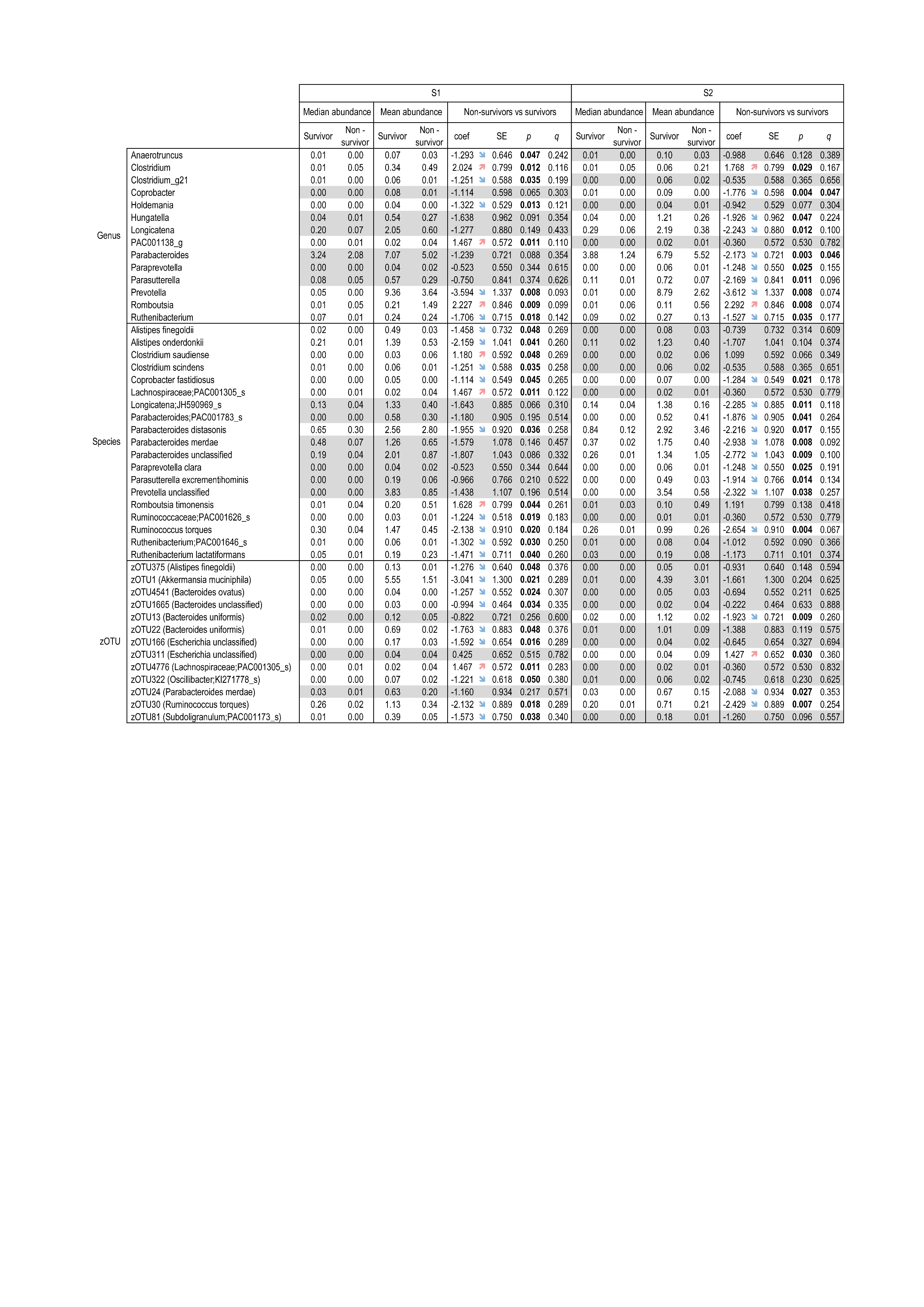


Legend: Results with an uncorrected *p*-value <0.05 (determined by MaAsLin2) observed in at least one sampling point are included. Bold values denote statistical significance in either uncorrected (*p*<0.05) or corrected data (*q*<0.05). Grey shading, indicating results without statistical significance before correction, is included for comparison purposes. Upward arrows indicate an increase (positive MaAsLin2 coefficient), while downward arrows indicate a decrease (negative MaAsLin2 coefficient) in relative abundance among non-survivors compared to survivors.

S1, first stool sample; S2, second stool sample; coef, MaAsLin2 coefficient; SE, standard error.

**Figure E2. Significant differences in relative abundance for genera between survivors and non-survivors**

Legends: S, survivors; NS, non-survivors; S1, first stool sample; S2, second stool sample. **p*<0.05

Bacterial genera showing statistically significant differences between survivors and non-survivors. Presented are results from MaAsLin2 with corrected *p*-values (*q*<0.05).

**Supplementary Table E3. Unadjusted Bayesian joint model analysis estimating the association between the change in gut microbiota diversity and mortality at D-60**

| Supplementary Table 1. Joint model analysis | | |
| --- | --- | --- |
| Estimating the association between the change in gut microbiota diversity and mortality | | |
|  | Estimate (95% credible interval) | P-value |
| Time-to-event | | |
| Decrease in Shannon index (Hazard ratio) | 1.94 (1.04 to 4.45) | <0.01 |
| Time-varying Shannon index | | |
| (Intercept) | 4.30 (4.00-4.60) | - |
| Change per day | -0.10 (-0.20 to -0.10) | <0.01 |
| Change per day * Antibiotics (from admission to S1) | 0.00 (0.00 to -0.10) | 0.03 |
| Change per day * Antibiotics (from S1 to S2) | 0.00 (0.00 to -0.10) | 0.13 |
| Sample size=96; Number of longitudinal observations=190; Number of events=24 (25%) | | |
| *p*-value represent the tail probabilities of containing the zero-effect value. | | |
